# AI Agents and Epidemic Intelligence on Respiratory Infectious Diseases: Toward a Conceptual Framework Integrating Decision Support

**DOI:** 10.2196/86936

**Published:** 2026-03-09

**Authors:** Liuyang Yang, Liyu Shan, Xiaolin Cao, Jinzhao Cui, Michael Tong, Yan Niu, Ting Zhang

**Affiliations:** 1 The Third Affiliated Hospital of Kunming Medical University, Yunnan Cancer Hospital, Peking University Cancer Hospital Yunnan, Kunming, Yunnan, China; 2 School of Population Medicine and Public Health, Chinese Academy of Medical Sciences & Peking Union Medical College, No. 9 Dongdan Santiao, Beijing, Beijing, China, 86 010-65120012; 3 School of Data Science, Fudan University, Shanghai, Shanghai, China; 4 Institute of Medical Information, Chinese Academy of Medical Sciences, Beijing, China; 5 National Centre for Epidemiology and Population Health, The Australian National University, Canberra, Australian Capital Territory 2601, Australia; 6 Public Health Emergency Center, Chinese Center for Disease Control and Prevention, Beijing, China

**Keywords:** artificial intelligence agents, respiratory infectious diseases, surveillance, risk evaluation, early warning, decision support

## Abstract

Traditional epidemic intelligence relies heavily on human epidemiologists for data interpretation and reporting, which makes it resource intensive, slow to respond, and vulnerable to variability in professional expertise. To overcome these limitations, we propose an expanded conceptual epidemic intelligence quadripartite framework that extends the classical trinity of (1) surveillance, (2) risk evaluation, and (3) early warning with a fourth pillar, (4) decision support and intervention optimization through AI agents. Acting as 24/7 digital epidemiologists, multiagent systems can integrate heterogeneous signals from multisource surveillance systems, conduct contextual risk evaluation and adaptive forecasting, generate tailored early warnings, and provide actionable recommendations for targeted control—closing the loop between detection and response. Embedding interpretability and mandatory human-in-the-loop oversight enhances trust and accountability. Nonetheless, real-world deployment requires addressing context-specific challenges of data quality, interoperability, robustness, governance, circular reporting, and equity. If designed with transparency, inclusiveness, and resilience, AI agents have the potential to transform epidemic intelligence into a continuously adaptive and globally connected system.

## Introduction

Respiratory diseases, such as seasonal influenza, respiratory syncytial virus (RSV), and emerging zoonotic respiratory pathogens, remain among the most significant causes of global morbidity and mortality. Seasonal influenza infects up to 1 billion people annually, causing an estimated 290,000 to 650,000 deaths each year [1], while RSV is responsible for approximately 33 million cases and 100,000 child deaths worldwide each year [2]. The rapid transmissibility, unpredictable evolution, and potential for interspecies spillover events make these pathogens a persistent global health threat. Recent outbreaks, such as the COVID-19 pandemic, underscore the urgent need for advanced epidemic intelligence. In particular, capturing the critical “golden window”— a short but decisive period during which timely detection and intervention can accelerate responses such as vaccine development and deployment—is essential to limit transmission, prevent missed opportunities for effective containment, and reduce the broader societal and economic impacts of respiratory infectious diseases.

Traditional respiratory disease surveillance systems, relying on clinician reporting, laboratory confirmation, and manual data collation, are often constrained by delayed signal detection and fragmented workflows. While event-based surveillance platforms, such as ProMED-mail and HealthMap, have integrated nontraditional signals, the transition from detection to actionable response remains suboptimal. Emerging artificial intelligence (AI)–driven frameworks, such as BlueDot [1] and the World Health Organization (WHO) Mosaic surveillance framework [2,3], demonstrate that fusing multisource signals with real-time analytics can substantially reduce analytical latency. However, technical acceleration alone cannot bridge the gap to intervention; the “golden window” is frequently missed due to governance bottlenecks, including bureaucratic friction and varying political will [4]. Consequently, there is an evolving need for orchestrated multiagent systems (MAS) that move beyond passive dashboards to provide active, evidence-based decision support, helping to navigate both data complexity and institutional inertia [5].

The “epidemic intelligence trinity,” a framework recently consolidated by the authors, comprises three functional stages: (1) surveillance and detection, (2) risk evaluation, and (3) early warning [6]. Surveillance aggregates heterogeneous outbreak indicators, risk evaluation contextualizes them to estimate potential spread and impact, and early warning translates these insights into alerts and intervention triggers. However, in current practice, these stages often remain linear and siloed. Information flows from surveillance to warning but often lacks the feedback loop required to optimize interventions immediately.

This Viewpoint presents a conceptual framework that advances beyond existing models by introducing “decision support” as a fourth, explicit pillar and using AI agents to drive the workflow. Unlike the WHO Mosaic framework, which primarily focuses on the diversity of data sources (“tiles”), or the traditional trinity, which emphasizes situational awareness, our quadripartite framework prioritizes autonomy and action. It proposes moving from passive dashboards to active MAS that not only display data but autonomously reason over it to suggest specific interventions, closing the loop between intelligence and response. Throughout this paper, we illustrate this potential through a recurring case scenario of a hypothetical zoonotic spillover to demonstrate tangible impacts.

**Figure 1.**
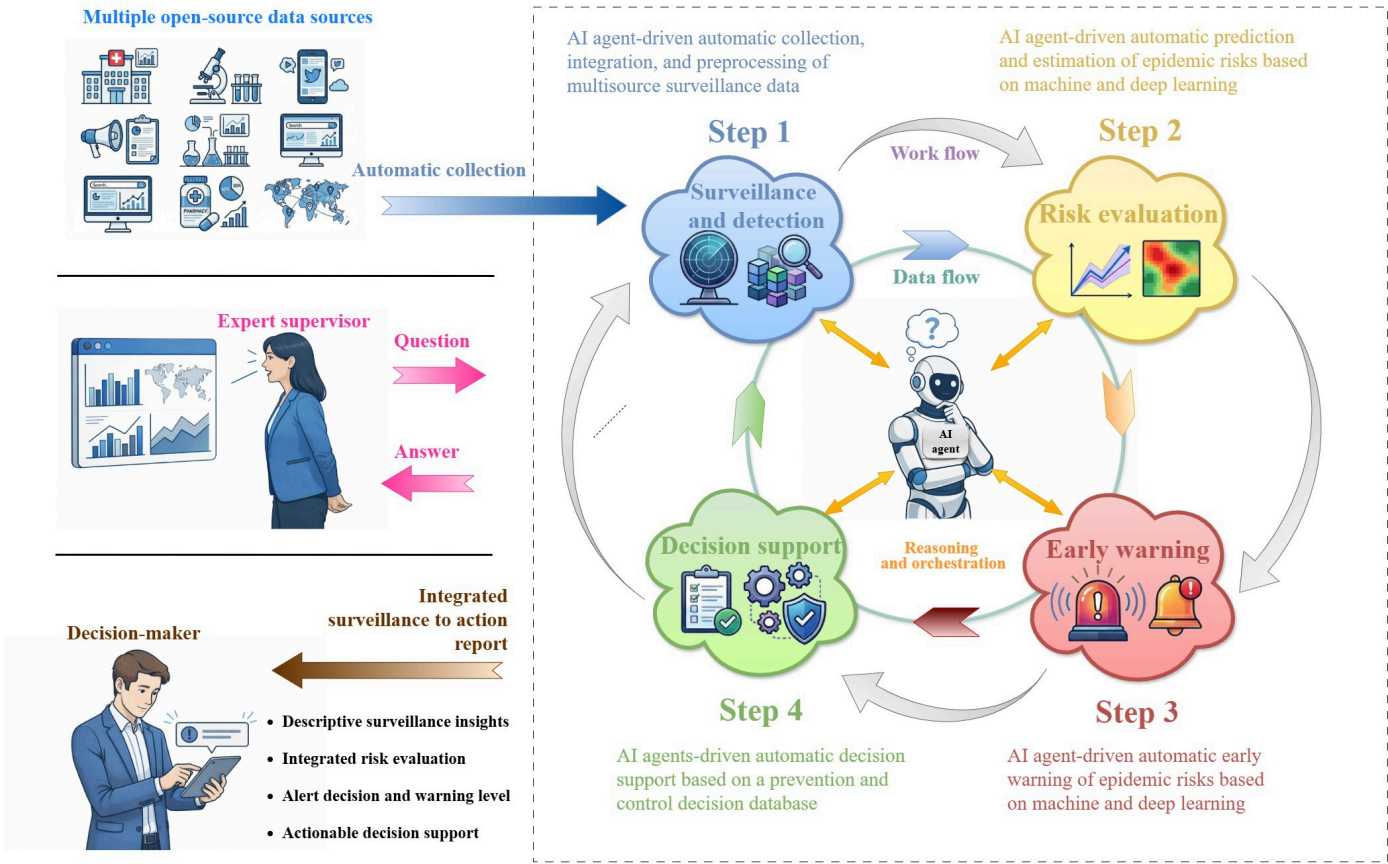
Artificial intelligence (AI) agents–driven integrated closed-loop framework for respiratory infectious disease surveillance, risk evaluation, early warning, and decision support. The framework illustrates an autonomous cycle for respiratory infectious disease management consisting of four interactive stages: (1) surveillance and detection—autonomous collection, integration, and preprocessing of multisource data; (2) risk evaluation—automated prediction and estimation of epidemic risk using machine learning and deep learning; (3) early warning—generation of timely alerts based on risk analysis; and (4) decision support—provision of targeted prevention and control recommendations through a decision database. At the core, the AI agent serves as the “brain,” performing reasoning and orchestration to manage data flow and workflow across these stages. The framework supports a hybrid interaction model: it primarily operates in a proactive mode by pushing integrated “surveillance-to-action” reports to decision-makers, while simultaneously supporting reactive queries from expert supervisors through an iterative question and answer process.

AI agents—autonomous computational entities capable of perceiving their environment, reasoning, and taking actions—offer a pathway to unify this process. Moving beyond static rule-based algorithms, modern AI agents use large language models (LLMs) to perform chain-of-thought reasoning and tool orchestration. This viewpoint examines how AI agents can enhance respiratory epidemic intelligence within this expanded framework (Figure 1).

## Defining AI Agents in Epidemiology

To understand the potential of this framework, it is necessary to define what constitutes an “AI agent” in this context. Unlike traditional deep learning models that passively process input to produce a prediction (eg, forecasting case counts), an AI agent is an autonomous system capable of three core functions.

Second, reasoning—using LLMs as a cognitive engine, the agent decomposes complex epidemiological problems into steps (chain-of-thought), interprets context, and plans tasks. For example, it can decide that a spike in “fever” search terms requires validation against pharmacy sales data.

Third, action—the agent executes tasks using external tools (application programming interface; API), such as running a transmission simulation model, drafting an alert email to health authorities, or querying a clinical guideline database.

In a MAS, specialized agents collaborate, handing off tasks to one another. This shift allows epidemic intelligence to move from a human-dependent “pull” system to an AI-driven “push” system, where the software proactively identifies threats and proposes solutions.

## Surveillance and Detection

Surveillance and detection constitute the first pillar of epidemic intelligence, aiming to identify early signals of outbreaks or abnormal disease activities. Traditional respiratory surveillance predominantly relies on single-source data, such as hospital reports of influenza-like illness (ILI), laboratory confirmation of pathogens, or clinician-reported unusual cases. While effective for confirmed cases, this passive and delayed approach may fail to detect emerging threats, particularly novel or geographically dispersed events.

In contrast, multisource “mosaic” surveillance integrates heterogeneous data streams to create a more sensitive and timely view of epidemic activity. The WHO has advocated a mosaic framework for respiratory virus surveillance, emphasizing that combining multiple “tiles” of data produces a more complete outbreak picture [2].

Given the complexity of diverse data modalities, an MAS approach is superior to a single monolithic agent. Specialized agents can autonomously ingest data from formal channels, such as EHRs, laboratory test results, pharmacy sales, and sentinel clinic reports, and from informal sources, including news media, ProMED-mail bulletins, social media trends, search index [7], participatory symptom reports, animal health bulletins, and environmental or climate datasets. Unconventional indicators, such as increased sales of cough or cold medication, spikes in fever-related online searches, and elevated SARS-CoV-2 RNA levels in wastewater, have been shown to precede clinical surges by days or weeks [8]. AI agents can continuously integrate and analyze such signals, functioning as tireless “digital epidemiologists.”

Traditional ILI surveillance has relied heavily on passive reporting, where clinicians manually record and submit case counts after aggregating data from their observations. This approach is inherently subjective, prone to underreporting, and often delayed. Increasingly, health systems are shifting to automated extraction of ILI indicators from EHRs, leveraging structured fields such as chief concerns, physical examination findings, and diagnostic codes. These systems can automatically flag and count encounters matching ILI case definitions in near real time.

Natural language processing further enhances surveillance by enabling AI agents to mine structured and unstructured data for outbreak clues. In addition to EHRs, agents can process informal text sources such as local news and social media [9], extracting disease-relevant facts, classifying signals by syndrome, and distinguishing credible reports from rumors. For instance, an agent might detect an increase in local language posts mentioning “pneumonia,” cross-reference this with wastewater testing data showing a coronavirus RNA spike, and confirm concurrent increases in web searches for “fever” and “cough.” The system can produce composite alerts with higher confidence and shorter detection lag than manual reporting or single-source monitoring by fusing such diverse signals. A critical advantage of AI agents lies in their ability to filter out noise while retaining sensitivity to subtle signals. In contrast, traditional systems often raise alert thresholds to avoid false alarms, which may result in missed events.

Moreover, AI agents can flag low-intensity anomalies and internally validate them against other data streams or reference knowledge bases. For instance, an agent detecting a sudden rise in emergency visits for respiratory illness could immediately check for similar patterns in nearby regions or seek plausible explanations, such as environmental events. The system would escalate an alert to human experts only when multiple indicators converge, thereby reducing false positives while preserving early warning capacity.

By automating and democratizing multisource data collection and analysis, AI agents extend surveillance to regions with limited public health infrastructure. This capability shortens detection lead times and reduces “blind spots” in low-resource settings, contributing to more equitable and timely global epidemic intelligence.

## Case Scenario

An AI agent designed for respiratory threats might continuously scan unstructured data. In a hypothetical scenario of a novel zoonotic spillover, the “surveillance agent” detects a statistically significant cluster of social media posts mentioning “severe cough” and “poultry die-off” in a specific province. Simultaneously, it cross-references this with real-time wastewater data showing an unidentified viral RNA spike in the same locality. Unlike a passive dashboard, the agent autonomously links these disparate signals and flags them for immediate processing. The practical superiority of these orchestrated systems in reducing analytical latency is further evidenced by recent empirical studies, which show significant improvements in lead time and sensitivity compared to traditional methods (Table 1 provides a detailed comparison).

**Table 1.** Comparison of traditional surveillance systems vs artificial intelligence (AI)–driven systems.

Study	Conceptual approach and data integration	Performance advantage (timeliness and accuracy)	Role in decision support and public health utility
Du et al [5]	LLM^a^-driven multimodal fusion: uses LLMs to synthesize heterogeneous data streams (news, reports, and clinical data) dynamically rather than static statistical modeling.	Real-time integration: outperforms baseline models in complex dynamics by processing unstructured data in real time.	Operational readiness and resource mobilization: provides rapid situational awareness to justify early resource allocation before clinical confirmation is available.
Idahor et al [10]	Paradigm shift (reactive to proactive): a theoretical framework contrasting rigid, siloed traditional reporting with fluid, big data–driven AI ecosystems.	Lag reduction: traditional systems lag by weeks; AI enables near–real-time monitoring by bypassing bureaucratic reporting delays.	Strategic planning and gap analysis: supports high-level policy shifts from reactive containment to proactive mitigation; identifies “blind spots” in existing infrastructure.
Xie et al [9]	Digital signal detection (social sensing): leverages LLMs to interpret social media sentiment and symptom reporting (Twitter [subsequently rebranded X, X Corp]) as a proxy for early epidemic signals.	Early warning (approximately 1 week lead): achieves approximately 7.63 days lead time over official epidemiological data; strong temporal correlation.	Preclinical alerting and risk communication: triggers public health messaging and targeted testing before hospitals see a surge, allowing for “flattening the curve” interventions.
Zhang et al [11]	Hybrid surveillance (environmental+clinical): integrates wastewater viral load monitoring with traditional clinical case reporting to form a dual verification system.	Leading indicator (0-7 days): wastewater signals precede clinical reports; combined systems show higher robustness than single-source methods.	Noninvasive community risk assessment: enables decision-makers to assess community transmission levels without mass individual testing; guides localized lockdowns or interventions.
Zhang et al [12]	Algorithmic anomaly detection: compares machine learning (supervised or unsupervised) against static statistical thresholds (ADTM)^b^ for outbreak detection.	Sensitivity enhancement: warnings issued approximately 5 days earlier. Unsupervised ML^c^ achieved 100% sensitivity vs 71% for traditional thresholds.	Automated triage and sensitivity tuning: reduces “missed alarms” for critical threats; allows officials to adjust sensitivity thresholds based on available response capacity.

a LLM: large language model.

b ADTM: algorithmic detection and threshold monitoring.

c ML: machine learning.

## Risk Evaluation

Risk evaluation is the second core function of epidemic intelligence in respiratory infectious disease, focusing on interpreting detected signals to determine an outbreak’s likelihood, severity, and potential consequences. While detection identifies unusual patterns, risk evaluation contextualizes them, distinguishing events requiring urgent intervention from those of limited concern [3].

Traditional risk assessments rely on expert review of epidemiological, clinical, and contextual information. However, manual processes can be slow, particularly when integrating heterogeneous datasets such as pathogen genomic sequences, case demographics, population immunity, mobility data, and health care system capacity. AI agents can accelerate this process by automating the collation, integration, and preliminary analysis of such datasets in near real time.

Modern AI agents can apply predictive models to estimate outbreak growth rates, reproduction numbers, and geographical spread, incorporating uncertainties from multiple data sources [13]. They can also use machine learning to identify populations or regions at higher risk based on vaccination coverage, comorbidities, demographic profiles, and environmental factors. For example, an agent might assess whether a novel influenza strain detected in wastewater coincides with low immunity levels in nearby populations, indicating a heightened potential for community spread.

Importantly, risk evaluation by AI agents is not limited to epidemiological modeling. AI agents can incorporate operational factors, such as testing capacity, supply chain status for antivirals, or intensive care unit (ICU) bed availability, to provide decision-makers with a comprehensive risk profile. However, it is important to acknowledge that obtaining real-time operational data is challenging due to the fragmented nature of health information systems and inventory databases. These assessments can be updated dynamically as new information becomes available, ensuring that public health authorities receive timely, data-driven insights to guide intervention priorities.

## Case Scenario

On receiving the signal from the surveillance agent, the “risk evaluation agent” automatically initiates a probabilistic assessment. It retrieves current population immunity data for influenza subtypes in the affected region, factors in local hospital capacity (ICU bed availability), and runs a transmission simulation. It calculates that the effective reproduction number (Rt) could exceed 2.5 within 7 days if left unchecked, classifying the event as “high risk.”

## Early Warning

Early warning aims to alert relevant stakeholders quickly enough for interventions to prevent widespread transmission [14,15]. Traditional systems often delay alerts until events are verified through official channels, which can reduce timeliness. AI agents can bridge this gap by issuing graded alerts based on probability thresholds, while continuing to refine assessments as new data become available.

An AI agent’s early warning process typically follows three steps: (1) aggregating multisource indicators into a composite risk score; (2) comparing the score against predefined alert thresholds; and (3) communicating the alert in structured, actionable formats. By using anomaly detection and probabilistic forecasting models, agents can provide both short-term outbreak forecasts and medium-term epidemic scenarios [16].

Unlike static rule-based systems, AI agents can adapt their alerting logic over time by incorporating feedback from both human experts and real-world outcomes, thereby creating a continuously improving feedback loop. In practice, this adaptation relies on human-in-the-loop review, where epidemiologists confirm or dismiss alerts, as well as retrospective validation once ground truth data become available. Alerts can be stratified by urgency; for example, an “advisory” for low-probability but high-impact threats and an “action alert” when confidence is high and immediate measures are recommended.

Dissemination channels can also be diversified: AI agents can automatically generate reports for public health dashboards, push notifications for field epidemiologists, and plain language summaries for community stakeholders. This ensures that early warnings reach the right audience in time to act, a critical factor in respiratory pathogen control where delays of even a few days can lead to exponential case growth.

## Case Scenario

The “early warning agent” takes the “high risk” classification and drafts a stratified alert. It generates a technical “action alert” for the local Centers for Disease Control, detailing the viral anomalies and projected trajectory. Simultaneously, it prepares a plain language “advisory” draft for community health workers, emphasizing vigilance for patients who have had contact with poultry. These drafts are held for human verification but are prepared instantly, saving hours of manual drafting.

## Decision Support

Decision support extends beyond warning, guiding the choice and timing of interventions to minimize health and socioeconomic impacts. Respiratory epidemic control includes recommendations for targeted vaccination, nonpharmaceutical interventions, antiviral distribution, and communication strategies.

AI agents can synthesize epidemiological forecasts, resource availability, and intervention effectiveness data to recommend optimal response strategies [4]. For example, given an emerging RSV outbreak in a region with limited ICU capacity, an agent might recommend targeted nonpharmaceutical interventions for high-risk populations, rapid deployment of prophylactic treatments, and public messaging to reduce exposure risk.

Given the severe safety risks associated with LLM hallucination (fabrication) in medical contexts, decision support modules must implement strict guardrails. Recommendations must be grounded in verified clinical protocols and subjected to retrieval-augmented generation techniques to ensure factual accuracy. Decision support also benefits from scenario simulation. AI agents can run counterfactual models to estimate their potential impact by comparing projected outcomes with and without specific interventions. These simulations can incorporate real-time updates, allowing response plans to adapt dynamically as the situation evolves.

Transparency is essential: decision recommendations must be explainable, clearly indicating underlying assumptions, data sources, and model limitations. This ensures that human decision-makers can assess the validity and acceptability of AI-driven guidance before it is implemented. In this role, AI agents act not as autonomous decision-makers but as analytical partners—augmenting human expertise with rapid, data-rich, and evidence-based insights that can shorten reaction times and improve intervention precision.

## Case Scenario

The “decision support agent” queries a database of evidence-based interventions. On the basis of the projected Rt and local resource constraints, it proposes a ranked list of interventions: (1) immediate targeted closure of live poultry markets in the identified district, (2) reallocation of antiviral stockpiles from the neighboring province, and (3) activation of a fever-screening protocol at local transport hubs. These recommendations are presented to human decision-makers with citations to relevant clinical protocols.

## Challenges and Practical Implementation Guidance

While promising, real-world deployment demands addressing specific challenges through concrete practical measures.

## Data Quality and Interoperability

AI agents depend on high-quality, diverse inputs. Informal sources may be noisy or biased, while data gaps in low-income regions risk reinforcing inequities, necessitating careful curation and cross-verification to prevent the system from optimizing primarily for data-rich regions. A critical risk is “circular reporting,” where agents amplify their own signals; therefore, rigorous source provenance tracking is essential to distinguish between organic signals and AI-generated content. To address data fragmentation, such as inconsistent formats in wastewater surveillance reports or a lack of standardized electronic medical record coding, implementation must adhere to open standards, such as HL7 Fast Healthcare Interoperability Resources and WHO protocols, to ensure seamless interoperability [12]. Furthermore, to overcome data sovereignty barriers, privacy-preserving technologies such as federated learning can enable global model training without transferring sensitive local data.

## Model Robustness and Validation

Complex models risk false positives or false negatives, potentially triggering inappropriate responses or missing critical threats. Rigorous validation is necessary, measuring lead time, sensitivity, specificity, and forecast accuracy against benchmarks. However, optimizing these metrics requires a delicate balance; an overly sensitive system risks generating “alert fatigue,” where a high volume of low-confidence warnings desensitizes human analysts. If operators are inundated with false alarms, they may eventually ignore genuine threats, rendering the surveillance system ineffective. Validating on historical data using internet-connected LLMs carries a risk of “look-ahead bias,” as these models may have “seen” the outcomes during training. Therefore, “prospective shadow testing” is recommended as a more rigorous alternative, where the agent runs in parallel to existing systems without releasing active alerts, allowing for safe evaluation of performance. Transparent, adjustable decision thresholds and iterative human-AI feedback loops can enhance reliability. Initial deployment should involve close human oversight, with gradual autonomy as confidence in model performance grows.

## Explainability and Trust

High-stakes decisions require explainable outputs. Explainable AI approaches can improve transparency by highlighting relevant data signals and causal rules. LLM agents can generate narrative rationales, but these must reflect actual reasoning to avoid conveying misleading confidence. Trust in AI agents will grow as they demonstrate value under expert supervision; however, high-profile errors could set progress back.

## Political and Bureaucratic Friction

Even the most precise AI-driven warnings may encounter friction as they move upward through institutional hierarchies. Decisions such as introducing lockdowns or prioritizing vaccine distribution are not solely forecasting problems; they are high-stakes governance and optimization problems influenced by economic concerns, political incentives, and competing narratives. AI agents must therefore be viewed not as a panacea for delay but as tools to provide clearer evidence-based support under radical uncertainty, potentially reducing, although not eliminating, the friction within political decision processes.

## Ethical and Legal Considerations

Privacy, consent, and accountability are critical concerns. AI agents must respect anonymity and avoid individual identification without consent. Legal frameworks for automated public health decisions remain underdeveloped, raising questions of liability in the event of errors. Human-in-the-loop governance must be framed not merely as a method for enhancing trust but as a mandatory legal and safety requirement before any “decision support” outputs are acted upon. Equity demands ensuring benefits for low-resource settings, and WHO-led global AI intelligence services could help provide access for all countries [17]. Furthermore, the economic sustainability of 24/7 digital epidemiologists must be considered, as continuous inference on large models is computationally expensive [18].

## Cybersecurity and Misuse

Adversarial attacks, such as false data injection, could disrupt or manipulate surveillance outputs. Systems must authenticate data and resist manipulation. Risks of misuse, including political exploitation for restrictive measures, necessitate algorithmic transparency, independent audits, and adherence to human rights norms.

## Workforce, Infrastructure, and Human-AI Interaction

Effective integration requires both skilled personnel and adequate infrastructure. Human epidemiologists should work alongside AI agents (digital epidemiologists) to validate outputs and enrich datasets. However, we must strictly mitigate the risks of “automation bias” and overreliance. If public health officials passively accept algorithmic outputs without scrutiny, it can lead to dangerous “misprioritization,” where resources are diverted based on high-confidence but erroneous model predictions. Furthermore, long-term dependence on automated agents risks a “loss of expertise,” where the workforce’s ability to independently interpret raw epidemiological signals and nuances degrades. Therefore, workflows must be designed to keep humans cognitively engaged—using AI as a “triage tool” rather than a replacement—and regular “manual mode” training simulations should be mandated to maintain core analytical competencies. Low-resource settings may rely on cloud-based services to host AI capabilities. Regular evaluations, including simulated outbreak drills, are necessary to test readiness and refine performance.

## Strategic Road Map

To transition this framework from concept to reality, we propose four priority actions: (1) standardization—develop global data dictionaries and API standards for respiratory surveillance to facilitate cross-border AI agent collaboration; (2) validation—establish standard “epidemic benchmark datasets” to rigorously test AI agent performance against historical ground truths; (3) pilot programs—deploy shadow-mode AI agents in sentinel surveillance sites to evaluate real-world utility and workflow integration; and (4) governance frameworks—draft international guidelines defining liability and accountability for AI-supported public health decisions.

## Conclusions

The convergence of advanced AI and the urgent need for improved epidemic intelligence offers a transformative opportunity. By extending the traditional trinity into a proactive loop with decision support, AI agents embody the “universal epidemic intelligence model” [7]. Through autonomous workflows, ranging from detecting weak signals in wastewater to recommending targeted market closures, agents can contextualize respiratory outbreaks days or weeks sooner than traditional methods. Realizing this vision demands investment, transparency, and equity, alongside the practical safeguards and road map outlined earlier. If implemented responsibly, these “force multipliers” can network globally to create a distributed immune system, unifying fragmented data into actionable knowledge—a capability essential in an interconnected world where pathogens ignore borders. The integration of AI agents into epidemic intelligence represents a transformative opportunity to enhance global health security. By addressing the outlined challenges and implementing the proposed road map, we can create a continuously adaptive and globally connected system. We call upon policymakers, researchers, and public health professionals to collaborate in realizing this vision, ensuring a more resilient future against respiratory infectious diseases.

## Abbreviations

AI: artificial intelligence
API: application programming interface
EHR: electronic health record
ICU: intensive care unit
ILI: influenza-like illness
LLM: large language model
RSV: respiratory syncytial virus
WHO: World Health Organization
